# In patients with acute myocarditis, the difference in apparent extracellular volume fraction between affected and healthy myocardium does not differ between early and late post-contrast imaging

**DOI:** 10.1186/1532-429X-18-S1-P246

**Published:** 2016-01-27

**Authors:** Magnus Lundin, Peder Sörensson, Peter Kellman, Andreas Sigfridsson, Martin Ugander

**Affiliations:** 1 Department of Clinical Physiology, Karolinska Institutet, Stockholm, Sweden; 2 Department of Molecular Medicine and Surgery, Karolinska Institutet, Stockholm, Sweden; 3 National Heart, Lung, and Blood Institute, National Institutes of Health, Bethesda, MD USA

## Background

Prior studies have shown that early post-contrast T1-weighted imaging can be useful for detecting global and focal relative enhancement abnormalities in myocarditis. We sought to determine whether early imaging (<5 minutes post bolus) affects the difference in apparent extracellular volume fraction (ECV) between healthy and affected myocardial segments in acute myocarditis.

## Methods

Consecutive patients referred for clinical 1.5T (Siemens Aera) cardiovascular magnetic resonance (CMR) evaluation of suspected heart disease were prospectively enrolled. T1 mapping was performed with a modified Look-Locker inversion recovery (MOLLI) sequence before and approximately 4, 10 and 20 minutes after an intravenous contrast bolus (Dotarem, gadoteric acid, 0.2 mmol/kg). Patients were included if the CMR findings showed acute myocarditis, and excluded if there were severe artefacts or if there were only affected or only healthy segments in the slice imaged over time. Segments were said to be *affected* if they had a native T1>1050 ms and *healthy* otherwise. Regions of interest for T1 measurement were placed midmurally in 6 myocardial segments in a midventricular short-axis slice and in skeletal muscle. Left ventricular blood pool T1* and venous hematocrit were measured. Apparent ECV was calculated for all time points post-contrast.

## Results

Patients (*n*=9, age 41 ± 19 years, 89 % male) had a mean ± SEM ECV 20 minutes post contrast of 26 ± 2 % in healthy and 32 ± 2 % in affected myocardium (*p*=0.001). The difference in apparent ECV between healthy and affected segments was 5.3 ± 1.0 % points 4 minutes after contrast, 5.6 ± 1.3 % points 10 minutes after contrast (*p*=0.69 vs 4 minutes), and 5.2 ± 1.1 % points 20 minutes after contrast (*p*=0.84 vs 4 minutes), see Figure. Furthermore, relative enhancement (ECV of myocardium divided by ECV of skeletal muscle) for the affected segments was 19 ± 3 % higher compared to healthy segments at 4 minutes, with no difference over time post-contrast (*p*=0.37 and *p*=0.56, respectively).

## Conclusions

In patients with acute myocarditis, early (4 minutes) and late (10 or 20 minutes) post-contrast imaging measurements do not differ with regards to quantitative differences in apparent ECV or relative enhancement between healthy and affected myocardium.

**Figure 1:**
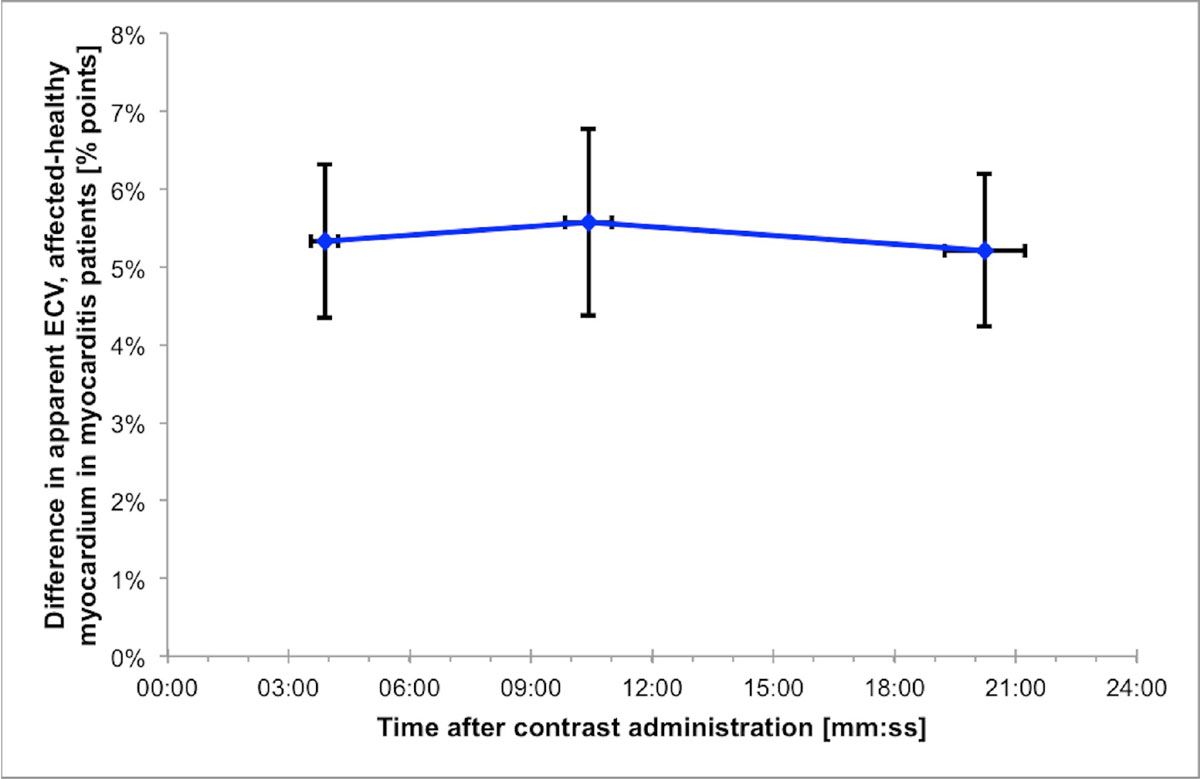
**Difference in apparent extracellular volume fraction (ECV) between affected and healthy myocardial segments over time post contrast administration in patients with acute myocarditis**. Data are given as mean ± SEM.

